# Living in the Shadows: Plight of the Undocumented

**DOI:** 10.1002/jclp.22361

**Published:** 2016-08-23

**Authors:** Roy Aranda

**Affiliations:** ^1^ Long Island Psychological Consulting

**Keywords:** asylum, undocumented immigrants, global mental health, cultural competence

## Abstract

The word “immigration” has become a household buzzword. The welcome sign on the Statue of Liberty that reads, “Give me your tired, your poor, your huddled masses yearning to breathe free,” however, is fading and has been replaced by many complicated conditions. What to do with the very large number of undocumented immigrants living in the United States and arriving at the United States every day commands considerable attention and has been the subject of breaking stories in the news. Working in the field of immigration demands an awareness of and sensitivity to diversity and cultural competence. Despite a “hot” sociopolitical climate when it comes to undocumented aliens and what to do with them, there are many ethical tenets that psychologists must be familiar with, among them rendering competent multicultural services. This article offers an overview of immigration law, the challenges of performing culturally competent assessments and consequences of failing to do so, and the plight of a particularly vulnerable group: unaccompanied children. Vignettes offer a personal look into the proceedings of 7 undocumented individuals in 4 major areas: asylum, hardship, U‐Visa, and VAWA.

## Introduction

Dolores, at the tender age of 21, wanted to escape from a life of sexual abuse at the hands of her stepfather. She fled from the Honduran small town her mother lived in to the much larger city, San Pedro Sula, and without telling a soul, ventured the many risks in the oftentimes perilous journey across Guatemala and Mexico to find safe haven in the United States. The journey, long and arduous, took 2 months. She traveled with several “coyotes,” the term for individuals who guide immigrants into the United States (WiseGeek, 2016). Along the way, she spent nights in shanty houses, took a multitude of transportation vehicles, and boarded the infamous “La Bestia” (The Beast, the name given to the huge cargo trains that traverse the length of Mexico carrying as many as 1,300 migrants) for a significant part of the way. Dolores had to run alongside the train at a point where it slowed down, grasped a rail, and climbed to the very top of the car that was loaded with “passengers” who were sitting or lying down. It was there where she was held up and robbed of her money and some belongings, leaving her quite shaky but still determined.

The final leg of the voyage required crossing the Rio Grande using an inflated plastic bag as a makeshift raft during the middle of the night in the pitch black, where, with the assistance of the last coyote, she stepped foot onto U.S. soil in the Rio Grande Valley. “Where to go?” Dolores asked herself. She followed a path worn by the footsteps of many others before her and, with trepidation, ventured forward not having a clue as to where she was going or how she would fare. After some walking, she saw a large vehicle with bright lights across the top in front of her, the sight of which made her heart pound with fear but also with hopeful anticipation. Moments later, Dolores found herself in the custody of Border Patrol agents. She was whisked away for processing, and with that she began what she hoped would be a new life free from sexual abuse in the Free World.

Dolores looked much younger than 21. And because her papers had been stolen when she was held up, she was able to convince immigration authorities that she was a minor. She spent 2 months in an immigrant youth detention center instead of the much larger jail‐like border adult detention centers. She told her story to the authorities and was able to initiate an asylum petition. This was very fortunate for her. Without legal representation, and not speaking or reading English or having any idea about immigration law, Dolores could have missed the 1‐year deadline to petition asylum and been subject to removal.

Dolores's case highlights just some of the conditions and reasons that prompt large numbers of Latinos to leave their countries, face many uncertainties, and live a life in the “shadows” marked by much stress and insecurity. Why would anyone make such a risk‐ridden decision? The belief that survival justifies facing the unknown, and the hope of a better life, weighed in favor of Dolores assuming the many risks that lay ahead. Of the many immigration‐related proceedings in the United States, Dolores's falls under what is known as asylum.

Immigration, and what to do with the very large number of people residing in the United States and arriving to the United States every day with undocumented status, has commanded considerable attention for decades and has increasingly been the subject of breaking news stories. We are at the cutting edge of major changes as many states already have enacted precedent‐setting legislation. On the horizon: the Dream Act, high‐skilled visas, driver's licenses for undocumented drivers, slowing down deportations, providing a mechanism of legalization for undocumented immigrants that does not lead to citizenship, and Deferred Action for Childhood Arrivals (DACA). There also have been recent changes in U.S. asylum laws.

Working in the field of immigration demands an awareness of and sensitivity to diversity and cultural competence. It is essential that the needs of undocumented immigrants and family members affected by their possible removal, asylum seekers, and victims of spousal abuse and domestic violence be adequately met, and that they not encounter untoward barriers to access needed services. And, consistent with the tenets of cultural and diversity‐based competence, it is imperative that those who work with these individuals receive proper training about the populations they serve, the conditions in their countries of origin, and the specific problems they face. This obligation also extends to interpreters, whose assistance is often required because of a language barrier.

This article provides an overview of immigration law, the challenges of performing culturally competent assessments and consequences of failing to do so, the plight of unaccompanied children, and several vignettes for a peek into the life in the shadows of a few undocumented–I abhor the word “illegal”–individuals I worked with in my private practice as a psychologist. I have evaluated and treated Latinos–documented and undocumented–for over three decades and have been qualified as an expert in immigration proceedings.

We are in a world in which culture and diversity are cornerstones of the health care industry, influencing the role of psychologists in multiple settings, including educational, clinical, forensic, research, and organizational settings. With this in mind, the challenge to our profession requires that we recognize, understand, and develop skill sets necessary to provide competent services that are consistent with our ethical standards and result in better outcomes, even as we navigate a sea of political uncertainty when it comes to undocumented aliens.

These principles and guidelines carve out basic ethical boundaries that psychologists are expected not to cross. There also are well‐defined cultural and diversity‐based components. Psychologists must, for example, be familiar with cultural differences and be aware of nuances stemming from age, gender, gender identity, race, ethnicity, culture, national origin, religion, sexual orientation, disability, language, and socioeconomic status. They must employ culturally sensitive and valid methods in their assessments and be aware of their own limitations. The challenge of becoming culturally sensitive and competent is a tall order, but one that cannot be ignored or minimized given that approximately 55.3 million Hispanics reside in the United States (Pew Research Center, 2016).

With immigration on the radar and the subject of a recent tug of war captured by headlines such as “Battle Lines,” the reality of the tens of thousands of children who have crossed and continue to arrive at the U.S. border, escaping unthinkable violence and other horrid conditions that prompted them to flee without adult companions, is a matter that cannot be swept under the rug; or saved like an unread article to ponder another day; or left ignored to settle itself, a default position so many routinely take (CNN Politics, 2014). Undocumented immigrants–children and adults–must cope with not only the trauma of their migration and leaving family and everything they know behind, but also untold uncertainties, barriers, and discrimination; racism; precious few resources and sources of support; and few rights.

Traumatic experiences and inhumane treatment is not the exclusive domain of other countries. In assessing the contributing factors affecting the lives of undocumented immigrants, we are confronted with a thorny challenge of determining how much is due to the migration and life experiences in the country of origin and how much stems from life experiences in the United States. In a depressed client, for example, to what extent is the depression due to his or her life experiences in country of origin, and to what extent does it result from life experiences in the United States? We need to consider that there may be an original or primary trauma as well as possible secondary traumas in the United States.

Aside from fairly obvious physical consequences (diseases, injuries, malnutrition, etc.), the emotional trauma and lingering emotional consequences are significant (e.g., trauma and stressor‐related disorders including PTSD; depressive disorders; anxiety disorders; substance‐related disorders; acquired neurocognitive disorder due to traumatic brain injury pursuant to abuse/injury). Psychologists may be called upon to provide professional services in various stages of the immigration process and in several contexts.

- Assessment needs to distinguish screenings, assessments in a particular context to respond to issues and questions raised in that setting, and more detailed comprehensive clinical evaluations and forensic evaluations.
- A screening can take place immediately after arrival to the United States and placement in detention center or shelter; and a screening can also take place later to address one or more needs or requirements.
- An intervention point can be defined as an assessment done at a particular point in time in a particular setting, geared to address first and foremost situation‐specific questions and needs.
- The assessment is critical as a starting point, and paves the way for treatments, interventions, referrals, and other clinical services that ideally will take place as a result, barring constraints such as time, access to providers, and financial or legal regulations.

The consequences stemming from culturally inadequate and incompetent evaluations and treatment are far‐reaching. Several potential errors may arise. These are driven by a lack of culturally sensitive awareness of symptom expression, a lack of awareness of the limitations of multicultural assessment practices, a lack of culturally sensitive assessment techniques, and a lack of culturally appropriate skills. A major difficulty is that assessment tools are not normed on the populations in which they are applied. Tools, therefore, may not be valid for the intended target populations.

Developing multicultural competence requires focusing the lens of culture in the many contexts noted above (American Psychological Association [APA], 2012). Yet becoming competent is an aspirational goal. There is a beginning and middle but no end. We are never fully “competent.” Striving to become more competent is a lifelong process.

## Immigration Basics

Immigration law is one of the most complex fields of law. Psychologists–and especially forensic psychologists–have recognized the imperfect “marriage” that exists between psychology and the law, one that has become more harmonious as psychology has come to play increasingly important roles in legal proceedings and as attorneys have come to recognize the significant contributions psychologists can make in many cases they work on.

To be clear, it is the attorney's responsibility to move their cases and to use any and all resources that they believe will be most helpful to their clients. Psychologists, through their work, play a role, and as such are but one piece in the total puzzle that makes up the attorney's case. It behooves psychologists, nevertheless, to do their homework and become familiar with the key aspects of the law in immigration‐related matters they become involved in. Below is a brief summary of the legal basics in several such proceedings.

### Asylum

There are five grounds that are considered in asylum determination: (a) race, (b) religion, (c) nationality, (d) membership in a particular social group, and (e) political opinion.

To qualify for asylum, an applicant must show past persecution or fear of future persecution based on one of these five enumerated grounds. Applicants must complete an I‐589 Application for Asylum and submit a supplemental statement in which they “tell their story” that forms the basis for the requested relief. Domestic violence represents a relatively new basis for asylum based on the constantly evolving category of “membership in a particular social group.”

Discrimination based on gender occurs in many Latin American countries. The *U.S. Department of State 2011 Human Rights Reports*, for instance, reveals widespread discrimination and sexual crimes against women in El Salvador, Honduras, Colombia, Guatemala, and Mexico (U.S. Department of State 2012a, b, c, d, e). As an example, the social group for an abused woman from El Salvador would be “Salvadoran women who have been victims of domestic abuse in which the government is unwilling or unable to protect the victim” (U. S. Department of Justice Office of the Attorney General, 2008). A growing number of these claims have been reported.

Winning asylum relief poses a tough slippery slope for the petitioner. Despite coming from countries that tolerate domestic violence, applicants must demonstrate to an asylum officer the egregious circumstances upon which they base their petition as well as a credible fear of persecution or torture and inability to find protection in their own country before their case can go forward.

### Hardship/Removal

To win a cancellation of removal case, an undocumented immigrant must prove that removal (previously, deportation) would result in an exceptional and extremely unusual hardship to spouse, child, or parent who is a U.S. citizen or lawful permanent resident.

This is a very high standard to meet. Any separation will result in some sort of hardship. But how, in a removal case, is the hardship so unusual or substantial as to merit satisfying the standard? Factors to consider are as follows:

- The age of the alien, both at the time of entry to the United States and at the time of application for suspension of deportation
- The age, number, and immigration status of the alien's children and their ability to speak the native language and adjust to life in another country
- The health condition of the alien or the alien's child, spouse, or parent and the availability of any required medical treatment in the country to which the alien would be returned
- The alien's ability to obtain employment in the country to which the alien would be returned
- The length of residence in the United States
- The existence of other family members who will be legally residing in the United States
- The financial impact of the alien's departure
- The impact of a disruption of educational opportunities
- The psychological impact of the alien's deportation or removal
- The current political and economic conditions in the country to which the alien would be returned
- Family and other ties to the country to which the alien would be returned
- Contributions and ties to a community in the United States, including the degree of integration into society
- Immigration history, including authorized residence in the United States
- The availability of other means of adjusting to permanent resident status (U.S. Citizen and Immigration Services)

Hardship applies to the U.S. citizen or lawful permanent resident, not to the person in the removal proceeding. The benchmark for physical presence is 10 years.

### U‐Visa

The U‐Visa is an available remedy for victims of crimes who have suffered substantial mental or physical abuse in the United States and are willing to assist law enforcement and government officials in the investigation or prosecution of the criminal activity. Thus, the U‐Visa offers protection to undocumented crime victims and facilitates the government's ability to investigate and prosecute any of a number of qualifying criminal activities that violate U.S. criminal law including, for instance, sex offenses and felonious assaults.

### T‐Visa

A T‐Visa gives temporary nonimmigrant status to victims of “severe forms of human trafficking” on the condition that they help law enforcement officials investigate and prosecute crimes related to human trafficking.  However, if the victim is younger than 18 years of age, the law does not require cooperation with police to obtain a T‐visa.

Eligibility requirements are as follows:

- Must have been a victim of a severe form of human trafficking
- Must be present in the United States, American Samoa, or the Commonwealth of the Northern Mariana Islands
- Must cooperate with law enforcement unless younger than 18 years of age
- Must show that he or she would suffer extreme hardship involving unusual and severe harm upon removal

### Violence Against Women Act (VAWA)

VAWA allows immigrant victims of domestic violence, battery, and extreme cruelty the opportunity to seek legal immigration status in the United States independently of the abusive U.S. citizen or lawful permanent resident.

### Special Immigrant Juvenile Status

This form of relief is available for certain undocumented children. The following are threshold requirements after which there will be additional requirements:

- The child has been declared to be dependent on a juvenile court.
- Reunification with one or both parents is precluded because of abuse, neglect, abandonment, or a similar basis.
- The child's best interest is not served by returning to country of origin.

## Unaccompanied Immigrant Children

### Scope of the Problem

Border crossings of unaccompanied children have increased dramatically since 2012. U.S. Customs and Border Protection reported that as of mid‐June of 2014, more than 52,000 children crossed the border, and approximately three quarters of them originated from Honduras, Guatemala, and El Salvador (U.S. Customs and Border Protection, 2016).

The horrors many endure on the way to “freedom” are too little known or understood. Families are devastated and torn apart. Many of these children are escaping unthinkable violence. The abuses and very high homicide rates, victimization of women and children (including rape), human trafficking (including sex trafficking, aka “sex slave industry”), labor trafficking, drug crimes, sequestrations, threats by “mareros” (gangs), atrocities committed against LGBTQ people, and other abominations against human rights prompt many to flee just to stay alive.

As described in my opening paragraphs, immigrants ride atop huge freight trains called La Bestia. Some die climbing the train or lose a limb when they fall under the train. These child refugees often suffer from a variety of medical problems and illnesses (e.g., dehydration and diarrhea) and are in urgent need of protective adult care and supportive supervision.

Children are normally held in detention facilities until they can be reunited with a family member or transferred to foster care‐type settings (for children younger than 12 years of age) or large and overcrowded detention shelters (for children older than 12 years of age), where they endure jail‐like conditions. Immigration detention center guards and others responsible for these young refugees may lack proper training about the populations they serve, the conditions in their countries, and specific problems they face.

### Emotional Impact

Children are more susceptible to the dangers of the world than adults, and unaccompanied children who face their situation alone are even more vulnerable. As psychologists, what role do we have in speaking for unaccompanied children, in protecting them, understanding their needs, safeguarding their rights as human beings, empowering them, tending to their mental health needs, and as consultants within a vastly complicated legal system (immigration and criminal when they are victims of crimes or involved in criminal activities)?

Psychologists may play an instrumental role in the Unaccompanied Alien Child Protection Act of 2005, which addresses the care and custody of unaccompanied alien children in the United States. Certainly, under one of the “Ps”—protection–of the Trafficking Victims Protection Reauthorization Act of 2013 (Title XII of the Violence Against Women Reauthorization Act of 2013), psychologists can play a robust role. And, in another “P”—prosecution psychologists play a role to the extent that psychological injury is assessed (U. S. Government Publishing Office, 2016).

### Assessment Considerations

The long, arduous, and dangerous journey to “freedom” poses remarkable challenges, obstacles, and potential trauma to these children along several stages. The following five‐stage assessment‐intervention model provides a guide for assessment, upon which interventions can be recommended and made.

#### The emotional trauma exposed to in the country of origin that prompted the decision to flee

The assessment has to be thorough and detailed. But it does not stop in the here and now; it must go back to “ground zero” for the child (Stage 1). What was his or her life like then and what prompted the decision to flee or escape?

#### The dangers exposed to on the way to “freedom” and consequential emotional trauma (Stage 2)

Can you imagine what it must have been like for little Pepito to ride La Bestia and have seen people fall off? Or for Joselito, whose flotation bag ripped and he went into the Rio Grande, in the dark, and held on to a piece of drift wood for dear life while crossing the snake‐like river? It is important not to ignore or speed through the “journey”; instead, one must ask very detailed questions. Crossing over, albeit brief, may have left a deep emotional impact.

#### The trauma incurred by being placed in overcrowded “jail‐like” detention facilities, which, although may have been brief, may leave enduring emotional scars (Stage 3)

Can you imagine how the little, unaccompanied girl in a detention center who had latched onto Maritza felt when the guard told Maritza to ignore her and pay attention only to her son? Or how Maritza felt when she asked for medication for her son who was ill and was told by the guard that it did not matter to him if the boy lived or died?  These children, already vulnerable and emotionally fragile, face the added burden of not knowing what lies ahead and what is to become of them.

#### By the time these children arrive at their destination in the United States, they remain emotionally fragile and vulnerable (Stage 4)

What lies ahead now? They have to adjust to a life in the United States taking into consideration cultural, language, and financial barriers as well as separation from family and reliance on new caretakers. These children must also cope with the emotional trauma brought on by uncertainty: Will they be allowed to remain in the United States? These worrisome thoughts will weigh heavily as their legal status–especially now in an emotionally charged political climate–is mired in a volatile and complicated system.

#### The emotional trauma of having to return to country of origin in the event that relief is not granted (Stage 5)

How exactly does one prepare a child for removal? How does one inoculate against the emotional trauma the child will incur? What sort of resilience treatment can be offered? What sort of removal plan can be established? What resources might be available? Who can be contacted?

There is no magical wand that will make a mental health professional competent to work with these minors. Considerable training and hands‐on work goes into this. Clearly, the clinician performing an assessment must be sensitive to the child's age and development, recognize that he or she may be emotionally frail or traumatized, and be able to work with the child to gain trust. It is difficult work, made even more so by the lack of properly normed assessment tools and the prevalence of negative stereotypes.

## Case Examples

### Asylum

#### Silvia

At the age of 21, Silvia escaped a brief relationship with a man who kept her locked in a room with no windows until she was so sick from an infection that she could not move. When Silvia met Guillermo, he was sweet and very attentive. Silvia was smitten with him, but despite her mother's admonitions, moved in with him after briefly dating.

The “honeymoon” ended soon enough. Guillermo drank a lot, degraded her, and told her she was “fea” (ugly) and that other women were better. He pushed her and repeatedly threatened her. He would not let her leave and told her that he owned her. Guillermo assaulted her sexually. Silvia feared he would hit her and so engaged in nonconsensual sex to placate him and avoid problems. Guillermo locked Silvia in a room whenever he went out. He threatened to hurt her and her family if she left him. Guillermo took Silvia to her mother's home only after she became very ill and was getting worse because he deprived her of medical attention. It was at this time, amidst his threats, that Silvia was able to escape and come to the United States. She was only 21 years old when she left her family behind.

Silvia and Guillermo originate from El Salvador, a country that criminalizes rape. The law, however, is not aggressively enforced, and rape and other sexual crimes are widespread and underreported (U.S. Department of State, 2012a). Silvia sought protection in the United States through a legal mechanism known as asylum, completed an I‐589 Application for Asylum, and submitted a supplemental statement, as required by law.

Silvia's case represents a relatively new basis for asylum based on the constantly evolving category of “membership in a particular social group.” Here, the social group would be “Salvadoran women who have been victims of domestic abuse in which the government is unwilling or unable to protect the victim” (U.S. Department of Justice Office of the Attorney General, 2008).

Silvia's psychological evaluation revealed symptoms of depression and anxiety, disturbed sleep and appetite, low self‐esteem, recurrent intrusive recollections of the abusive relationship, and feelings at times that things are unreal. Not uncommonly, women like Silvia suffer from major depressive disorder (MDD) and posttraumatic stress disorder (PTSD). Silvia's PTSD was in partial remission, largely due to the curative effect of living with an uncle who had sponsored her. But emotional scars run deep, and recovery will be a long process.

Discrimination based on gender occurs in many Latin American countries. Some examples are as follows:

- Honduras: The *2011 Human Rights Report* indicated that “violence against women and impunity for perpetrators continued to be serious problems. The National Criminal Investigation Division reported it was investigating 3,148 complaints of domestic abuse and 473 killings of women (of more than 6,723 male and female victims of homicide)” (U.S. Department of State. 2012b).
- Colombia: The *2011 Human Rights Report* estimates that “489,687 women had been victims of sexual violence. Almost 20 percent of these were instances of rape; almost half of these women were raped at least twice (33 percent on three or more occasions)” (U.S. Department of State, 2012c).
- Guatemala: The *2011 Human Rights Report* indicated that “rape and other sexual offenses remained serious problems. According to the Public Ministry, 3,922 cases of rape were reported during the year. At year's end 10,526 additional cases of sexual abuse and other forms of physical violence were reported, according to the judiciary, PNC, and Institute of Public Defense” (U.S. Department of State, 2012d).
- Mexico: The *2011 Human Rights Report* indicated that “state‐level laws sanctioning domestic violence are weak. Seven states do not criminalize it, and 15 states punish it only when it is a repeated offense.” Victims do not report abuse because of fear of spousal reprisal, shame, and the belief that a complaint was not warranted. According to the 2006 National Survey on Household Relationships, 67% of women older than 15 years of age had suffered some abusive treatment. According to the Citizen Femicide Observatory, more than 1,700 girls, teenagers, and women were killed between January 2009 and June 2010 (U.S. Department of State, 2012e).

These statistics suggest that Silvia's plight is repeated far too many times and in too many places.

#### Nereida

30‐year‐old Nereida escaped to the United States fearing for her life after her older sister was brutally killed near their hometown in Honduras, a place that had witnessed 11 femicides in 1 month. The matter of femicide in Honduras is well documented (Femicide, 2011; Honduras’ Commission against Femicide, 2011). Nereida, like Silvia, suffered from clinically significant symptoms of anxiety and depression. She cried during the psychological evaluation and conveyed her fear that she would be killed like her sister was if she returned to Honduras. She had flashbacks of a local newspaper picture of her deceased sister and experienced a sense of loss of control in her life. And like Silvia, Nereida was hypervigilant, constantly looking over her shoulder, fearing that she was being watched or followed.

#### Marisol

25‐year‐old Marisol fled from her abusive husband in El Salvador because she feared for her life. She had been beaten numerous times and went to the police after he held a machete to her throat and threatened to kill her. He was detained for 3 days. He found her on the street after he was released and ran her over with his car. Marisol was hospitalized for several months and was confined to a wheelchair for half a year. Marisol hid at a friend's home. When he found her, he threatened her at gunpoint, demanding that she better return with him. Marisol did, and the next day, while he was out, she fled the country, leaving her two young children behind. Her diagnosis was MDD and PTSD. Neuropsychological testing also revealed a number of neurocognitive impairments stemming from the pedestrian–motor vehicle accident.

### Hardship

#### Carla

Carla is a 35‐year‐old woman who originates from the Dominican Republic and has been in the United States for approximately 6 years. She lives with Sonia, a U.S. citizen, whom she legally married in New York.

Sonia does not perceive moving to the Dominican Republic to be an option.She worries about Carla's well‐being because of high crime and violence in the Dominican Republic, and especially, hatred, discrimination, and violence toward people who are lesbian, gay, bisexual, and transgender (LGBT).

According to the Human Rights First (2015):

LGBT Dominicans face a range of human rights concerns including violence, discrimination, hate crimes, lack of access to justice, impunity for perpetrators, and societal homophobia and transphobia. …A police regulation criminalizes same‐sex sexual activity among the police force and the Dominican government does not allow same‐sex marriage. The country also lacks comprehensive nondiscrimination and hate crime laws. …LGBT Dominicans face the threat of violence and discrimination because of their sexual orientation and/or gender identity. …Lesbians and transgender people also face the threat of gender‐based violence and so‐called “corrective” rape. LGBT Dominicans also experience discrimination in accessing services including housing, employment, education, and healthcare. …Many do not report violations due to lack of trust in the police and justice system. In many cases, impunity is the norm. (Human Rights First, Hope Will Prevail: Advancing the Human Rights of LGBT People in the Dominican Republic, December, 2015)

Additionally, Sonia is scared that Jose, Carla's significant other in the Dominican Republic who was so threatening and abusive, will find her and hurt her–a possibility that would become even more likely if he were to learn that she is married to a woman. Reportedly, Carla knows through her mother and a friend that Jose asks about her. Another source of worry for Sonia is that there is no job waiting for Carla and employment opportunities in the Dominican Republican are meager. Moreover, Sonia and Carla have been together for a long time and Carla's departure would be damaging to their marriage, as well as having a deleterious effect on Sonia's already vulnerable emotional and physical well‐being.

#### Carlos

50‐year‐old Carlos originates from Ecuador and has been in the United States since 1999. He lives with his wife, Yvonne, and their two sons. Carlos suffered an occupational injury and has undergone several surgeries, most recently a cervical surgery that has left him with a cervical plate and four screws. He has a diagnosis of MDD, severe, and postconcussion syndrome; he is in treatment with a neuropsychiatrist and takes multiple medications.

Yvonne suffers from gastritis and mild scoliosis, is being treated for an ovarian cyst, and takes several medications. The older son, Jaime, suffers from several medical conditions, including chronic back pain, acute gastritis, abdominal pain, irritable bowel syndrome, and high liver functions. The younger son, Donny, presents with a history of speech and language impairment and special education.

Carlos worries about his legal situation and how his removal would affect their lives. He feels depressed and anxious. He is very troubled because he is sick and can't work and he is afraid of what will become of his family. He does not sleep well, has nightmares, and at times wakes up screaming. He presents with numerous somatic complaints stemming from his occupational injury.

Yvonne reported that she feels nervous because of her husband's legal situation. She worries for her family and is scared of being left by herself to tend to their two sons with medical issues and being without her husband's presence and emotional and financial support. Yvonne does not perceive moving to Ecuador to be an option because of her health problems and Donny's special needs and Jaime's health problems. She worries about her husband's well‐being because of high crime and violence in Ecuador (Ecuador Crime and Safety Report, 2015). There is no job waiting for Carlos who has been out of the country for approximately 17 years. Furthermore, as noted, Carlos has severe, chronic medical problems that would not be tended to in Ecuador because he has no insurance.

Yvonne would lose a husband and the children would be raised without a father. She does not work and the family depends on his Workers’ Compensation benefits. Their financial situation would be precarious. Donny has special education needs and is very attached to his father. Losing his father would be a great source of emotional distress and would have a deleterious effect on his socioemotional and educational development.

### U‐Visa

#### Alma

Alma is a 25‐year‐old woman who originates from El Salvador and has been in the United States for 8 years. Alma reported that she was attacked by a woman while working as a waitress at a bar in Long Island, New York. She stated that the other woman thought she was going out with the DJ and attacked her. In the attack, Alma's face was scratched and she was thrown to the ground and kicked. Alma's attacker was arrested and prosecuted with Alma's assistance, as required to petition a U‐Visa. Alma presented with a constellation of symptoms consistent with a diagnosis of PTSD, chronic.

### VAWA

#### Rosa

Rosa is 38‐year‐old woman who originates from Peru. For the past 2 years, she has lived with her sister in Long Island. Previously, she lived in Staten Island with her husband for one and a half years.

Rosa met her husband, Pablo, at work. They maintained contact for 2 years and were married 4 years ago. She stated that the relationship was strained early on. He did whatever he wanted and placed severe restrictions on her. She had to do whatever he wanted to avoid problems and was subjected to his orders all the time. She did things she didn't want to do just to appease him. He was emotionally abusive and frequently insulted and belittled her.

Her sister witnessed the verbal abuse and saw Rosa crying a lot. Rosa kept to herself and didn't want to get up and do things. She stayed in her room. She needed to be reminded to bathe and do things. She was emotionally labile and prone to mood swings. She dwelled a lot on the marriage and the bad things that Pablo did to her. Rosa's mood was depressed and she was soft‐spoken. She was tearful during the examination and reported suicidal ideation. She complained of poor sleep and frequent nightmares. She reported flashbacks related to the relationship and somatic complaints and headaches. She felt uncomfortable in social settings and insecure around people. She reported being unable to stop worrying and feared having a nervous breakdown. At times, she felt that things were unreal. Rosa's diagnosis was MDD.

A petition for asylum, based on VAWA principles was approved, supported by the fact that Rosa's husband subjected her to extreme emotional cruelty while they lived together.

## Conclusion

The field of psychology is rich and boasts a long history with many areas of specialization and subspecialties. Psychologists are required to abide by the 2010 Amendments of the *Ethical Principles of Psychologists and Code of Conduct* (APA, 2002a). These standards provide for minimal competencies. The *Specialty Guidelines for Forensic Psychology*, released in 2011, on the other hand, delineates a broad spectrum of aspirational guidelines for psychologists who perform work in forensic contexts (American Psychologist, 2012). These principles and guidelines as a whole carve out basic ethical boundaries that psychologists are expected not to cross.

There also are well‐defined cultural and diversity‐based mandates. Psychologists must, for instance, be familiar with cultural differences and be aware of nuances stemming from age, gender, gender identity, race, ethnicity, culture, national origin, religion, sexual orientation, disability, language, and socioeconomic status. They must employ culturally sensitive and valid methods in their assessments and be aware of their own limitations. The *Guidelines on Multicultural Education, Research, Practice, and Organizational Change for Psychologists* (APA, 2002b) also is not mandatory. Like the *Specialty Guidelines for Forensic Psychology*, these guidelines may be viewed as aspirational and seen as a work in progress that evolves over time. Cultural competence involves an ongoing process of self‐search, education, and practice.

As complex as the field of psychology is, imagine how much more complicated things can become when psychologists undertake forensic roles or perform work that combines psychology and law. The law is just as complex–perhaps even more so–than psychology. And in an area as difficult as immigration law, where legal regulations change, are expanded, and evolve as needs change in response to the perpetually changing state of migration, it behooves psychologists immersed or interested in this field to know the law well.

Working in the field of immigration calls for the highest awareness of and sensitivity to diversity and cultural competence. It is imperative that those who work with these individuals receive proper training about the populations they serve, the conditions in their countries, and specific problems they face. This article provided an overview of immigration law, the challenges of performing culturally competent assessments and the consequences of failing to do so, the plight of unaccompanied children, and brief narratives into the lives of several undocumented immigrants I evaluated in my private practice as a psychologist in four major areas: asylum, hardship, U‐Visa, and VAWA.

The needs of the tens of thousands of undocumented adults and children who have crossed and continue to arrive at the U.S. border to escape unthinkable violence and other horrid conditions cannot be ignored. Notwithstanding the friction that may exist between the law and those who heed the call to render humanitarian aid and assistance, psychologists can fulfill key invaluable roles in many contexts and applications that apply to "illegal" immigration.
